# Release of Tenofovir from Carrageenan-Based Vaginal Suppositories

**DOI:** 10.3390/pharmaceutics6030366

**Published:** 2014-07-04

**Authors:** Toral Zaveri, John E. Hayes, Gregory R. Ziegler

**Affiliations:** 1 Sensory Evaluation Center, the Pennsylvania State University, University Park, PA 16802, USA; E-Mails: tzz1@psu.edu (T.Z.); jeh40@psu.edu (J.E.H.); 2 Department of Food Science, College of Agricultural Sciences, the Pennsylvania State University, 341 Food Science Building, University Park, PA 16802, USA

**Keywords:** microbicide, tenofovir, dissolution, semisoft suppository, carrageenan, HIV

## Abstract

Microbicides are an active area of research for HIV prevention, being developed as a woman-initiated method of prevention during unprotected coitus. Along with safety and efficacy, assessing and improving compliance is a major area of research in microbicide development. We have produced microbicide prototypes in the form of semisoft vaginal suppositories prepared from carrageenan and conducted both qualitative and quantitative studies using these prototypes to determine the physical properties that drive acceptability and possibly adherence. In order to ensure that the suppositories function as effective drug delivery vehicles, we have conducted *in vitro* dissolution studies in water, vaginal simulant fluid (VSF) and semen simulant fluid (SSF) with suppositories loaded with the antiretroviral drug, tenofovir (TFV). TFV was released via diffusion and matrix erosion in water or by diffusion out of the matrix in VSF and SSF. Diffusion studies were conducted in two different volumes of VSF and SSF. The volume of VSF/SSF into which TFV diffused and the size of the suppositories determined the rate of diffusion from the suppositories. About 45%–50% of the encapsulated TFV diffused out of the suppositories within the first two hours, irrespective of suppository size, diffusion medium (VSF/SSF) and the volume of medium. Prior work indicates that a short waiting period between insertion and coitus is highly desired by women; present data suggest our microbicide prototypes have rapid initial release followed by a slow release curve over the first 24 h.

## 1. Introduction

In 2011, 34 million people worldwide were living with HIV [1]. Although there has been a significant decrease in the number of new HIV infections over the past decade [1], AIDS and other sexually transmitted infections continue to burden the global economy. Condoms have been successful in preventing the transmission of HIV [2] and other sexually transmitted infections [3]. However, effectiveness depends on correct and consistent use by the male partner [4]. Due to socioeconomic and gender inequities, women in some countries and cultures are not always in a position to negotiate regular condom use [5]. A microbicide is a product containing agents known to prevent transmission of HIV and other viruses that can be inserted into the vagina prior to intercourse in the form of a gel, cream, foam, sponge, suppository or film [6]. It is a woman-initiated prevention method that can protect against transmission of HIV and other sexually transmitted infections during heterosexual intercourse with a partner whose infection status may or may not be known to the woman [6]. In the United States, transmission through heterosexual intercourse accounted for 80% of the new HIV infections among women from 2006 to 2009 [7].

The antiretroviral drug tenofovir (TFV), delivered as a vaginal gel (1%), has been investigated in several microbicide clinical trials for prevention of HIV and Herpes Simplex Virus-2 (HSV-2) transmission [8]. The CAPRISA 004 study, investigating effectiveness of 1% TFV gel, demonstrated that the effectiveness of HIV prevention depended on the adherence to gel use; women reporting over 80% adherence showed a 54% decrease in HIV incidence, compared to a 28% decrease for the low adherence (<50%) users [9]. Thus, user adherence is a critical factor governing microbicide success, and various studies have been conducted to understand product attributes that influence acceptability and adherence [10,11]. These product attributes include appearance [12,13], smell [12,13], taste of the microbicide, textural properties that may affect sexual pleasure (how the product feels during intercourse) [11,14] or leakage (the propensity of the product to seep out of the body) [10,12,14,15], as well as vaginal coating [14]. Since microbicides are a coitally-associated product, women’s preferences for the product attributes may vary based on their sexual practices, as well as vaginal product usage. To address this need for different preferences, different types of microbicides are in development or clinical trials, including vaginal gels [9], intravaginal rings [16,17], tablets [18] and quick dissolving films [19,20]. Some negative attributes have been reported for these existing technologies; for example, leakage has been cited as one of the drawbacks of liquid gels [10,21] and tablets are hypertonic. To explore the intermediate design space between liquid gels and solid tablets, we have developed microbicide prototypes between these two extremes using a soft-gel technology. In our delivery system, semisoft vaginal suppositories are prepared from carrageenan, which has several advantages over gelatin, the traditional soft-gel matrix.

Carrageenan has been investigated for various biomedical applications, such as drug delivery [22], wound dressings [23] and microbicides for the prevention of HSV-2 [24] and papilloma virus [25]. Carraguard, a carrageenan-based gel, has been previously explored as a microbicide for HIV prevention; however, in spite of being well tolerated, further development efforts were discontinued due to its inefficacy in preventing HIV transmission [26]. Using carrageenan as the drug carrier in our semisoft suppository preparation, we aimed to circumvent the negative aspects associated with gelatin, such as the risk of zoonotic infections, the lack of acceptability by vegetarians and the insufficient heat stability for storage in tropical climates, as well as taking advantage of the anti-viral activity reported for carrageenan against viruses other than HIV [24,25,27]. We have previously conducted acceptability studies with our semisoft vaginal suppositories and identified physical attributes (size, shape and firmness) and user preferences (the frequency of application and the duration of protection) most favorable for women [13,28,29]. To be biologically efficacious, antiretroviral drugs must first diffuse out from the carrier matrix within a reasonable time in the vaginal environment, including exposure to semen. The physiological fluids present in the vagina modulate the drug release from carriers, act as a barrier for drug diffusion into vaginal epithelium, as well as cause product dilution [30]. The ions present within the vaginal and seminal fluid are of particular concern in our system as cations, such as K^+^ and Ca^2+^, affect carrageenan gelling. Delivery of tenofovir in a carrageenan-based gel has not been tested previously; hence, determining that the tenofovir is not bound irreversibly to the carrageenan matrix, but is released as a critical step in the development of the prototypes. Furthermore, it is important to characterize the drug release in conditions simulating the vaginal environment. The current study was designed to investigate the release kinetics of TFV from the semisoft suppositories in water, vaginal simulant fluid (VSF) and semen simulant fluid (SSF) *in vitro* to further the development of an effective microbicide.

## 2. Experimental Section

### 2.1. Materials

A commercial sample of κ-carrageenan (Gelcarin NF 911, Batch Number 10707011) was kindly provided by FMC Biopolymers (Philadelphia, PA, USA). The κ-carrageenan was converted to the sodium salt form by ion exchange using Amberlite IR-120 Na ion-exchange resin (Alfa-Aeser, Heysham, UK). Tenofovir (TFV) was kindly provided by Gilead Sciences (Foster City, CA, USA). All other reagents were purchased from VWR International (Bridgeport, NJ, USA) and used as received.

### 2.2. Suppository Preparation

**Figure 1:**
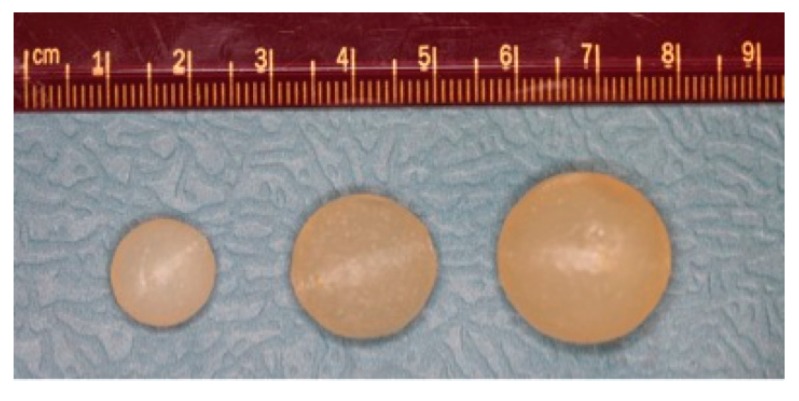
Spherical semi-soft vaginal suppositories, 1, 3, 5 mL (left to right).

Based on the dose of TFV delivered during microbicide clinical trials with 4 mL of 1% TFV gel [9], each suppository in our study contained 40 mg of TFV, irrespective of the size. The TFV was dissolved in deionized water, adjusted to pH 7.2–7.4 by the addition of sodium hydroxide. The highly acidic pH of TFV in water (pH 3–4) disrupted carrageenan gel formation, hence the need to adjust the pH towards neutrality. TFV solutions of varying concentrations were prepared in order to incorporate 40 mg TFV in suppositories of volumes of 1, 3 and 5 mL. To prepare the gels for semisoft suppositories, κ-carrageenan (3% (*w*/*v*)) and potassium chloride (0.025 M) were dissolved in the TFV solutions, followed by heating at 80 °C for at least 1 h to form homogenous dispersions. Prior to preparing the suppositories, the dispersion was characterized in a rheometer (described below) to ensure that the storage modulus matched the desired value of G’ = 25,000 Pa at 25 °C. Previous qualitative and quantitative studies performed by our group established that this firmness level is most preferred by women for vaginal suppositories [13,28,29]. These studies also established that a long oval shape was most preferred; however, spherical suppositories were used here to enable use of standard models of diffusion and to simplify diffusion rate calculations. Spherical suppositories in 3 sizes (Figure 1) were prepared by filling plastic syringes with the hot gel and injecting it into acrylonitrile butadiene styrene molds, followed by cooling in a refrigerator (4 °C) for 15 min to allow the gels to set.

### 2.3. Rheological Characterization of Gel

Rheological measurements were performed on the gel by subjecting it to small deformation oscillatory measurements on a strain-controlled rheometer (ARES, TA Instrument, New Castle, DE, USA) using a cone and plate geometry (probe diameter = 25 mm, cone angle = 5.73°). The gel was loaded between the plates when hot and the edges sealed with a light coating of mineral oil to avoid water evaporation. Spectra were recorded on first cooling followed by heating the gel at 5 °C per minute between 60 and 15 °C at a frequency of 1 Hz and a strain of 1%. The gel had a melting point above 50 °C, which is important for stable storage in hot climate areas.

### 2.4. Characterization of Drug Release

To ensure that the carrageenan-based suppositories would serve as functional drug delivery vehicles, *in vitro* dissolution studies were performed and the rate of release of TFV from the semisoft suppositories calculated. Dissolution studies were performed (1) using USP apparatus 2 (basket apparatus) with 100 mL glass vessels or (2) in a shaking incubator with 50 mL tubes. Studies were run at 150 rpm, 37 °C with 80 mL deionized water, VSF or SSF for the basket apparatus and 5 mL VSF/SSF for the shaking incubator. Dissolution studies with 5 mL VSF were designed to better mimic the vaginal environment, as the average volume of vaginal fluid obtained from healthy donors has been shown to vary in the range of 0.5–8 mL/day [31,32,33]. The volume of semen produced during ejaculation is shown to vary from 2 to 4.5 mL [34,35]. However, to compare the dissolution in VSF with SSF, a volume of 5 mL was selected for the SSF dissolution studies. The vaginal and semen simulant fluid was prepared as described by Owen and Katz [36,37].

The TFV released into the water, VSF or SSF during the dissolution studies was quantified by measuring absorbance at the peak maximum (260 nm) in the UV spectrum of TFV using Multiskan Go (Thermo Scientific, Vantaa, Finland), UV-Vis plate spectrophotometer. Absorbance values were converted into TFV concentrations using a standard curve of TFV in water (*r*^2^ = 0.985), VSF (*r*^2^ = 0.99) and SSF (*r*^2^ = 0.994) (0.125–0.0025 mg/mL).

The diffusivity of TFV was calculated using the following Equation (1):

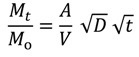
(1)
where *M_t_* = mg of TFV at time *t*, *M*_o_ = initial amount of TFV (40 mg), *A* = surface area of sphere (m^2^), *V* = volume of sphere (m^3^), *D* = effective diffusivity (m^2^ s^−1^), *t* = time (s).

### 2.5. Statistical Analysis

Data were analyzed using JMP v9.0.2 (Cary, NC, USA). For the TFV release data in water VSF and SSF, mean and standard error were calculated (*n* = 7) for each time point. For comparing the initial rates of diffusion (0–2 h) across different VSF and SSF volumes and sizes, the slopes for individual samples were computed from the release data. The least square means (LSM) of slopes were calculated and compared using Tukey’s HSD test with *p* < 0.05 considered significantly different.

## 3. Results

### 3.1. Release of Tenofovir (TFV) in Water

**Figure 2:**
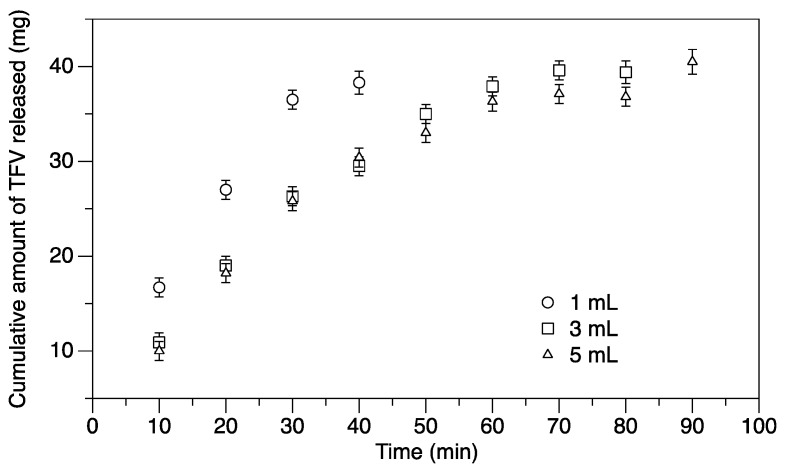
The effect of size on the release of tenofovir (TFV) from semisoft spherical suppositories in water. Release of TFV by matrix erosion from suppositories of 1, 3 and 5 mL loaded with 40 mg TFV each was studied, until the suppositories were fully eroded. Plotted are the mean and standard error (*n* = 7) of the cumulative amount of TFV released.

The carrageenan gel matrix eroded in water leaving a viscous fluid as the potassium ions holding the carrageenan helices together diffused into the surrounding fluid. As the matrix broke down, the TFV encapsulated within it was released. One milliliter spheres required about 40 min to completely dissolve and disperse the TFV as compared to 3 and 5 mL, which follow similar dissolution curves, taking 80–90 min to dissolve completely (Figure 2). Based on the cumulative release data, the loading efficiency of the ovules is 99%, and the drug is uniformly distributed in the carrageenan matrix.

### 3.2. Release of TFV into Vaginal Simulant Fluid (VSF) and Semen Simulant Fluid (SSF)

Due to the high cation concentration in the VSF and SSF, the carrageenan matrix did not erode as it did in water. Nonetheless, the TFV was released into the surrounding VSF or SSF media via diffusion. The effective diffusivity of TFV from the carrageenan matrix into VSF was calculated to be 4.2 × 10^−10^ m^2^ s^−1^.

**Figure 3:**
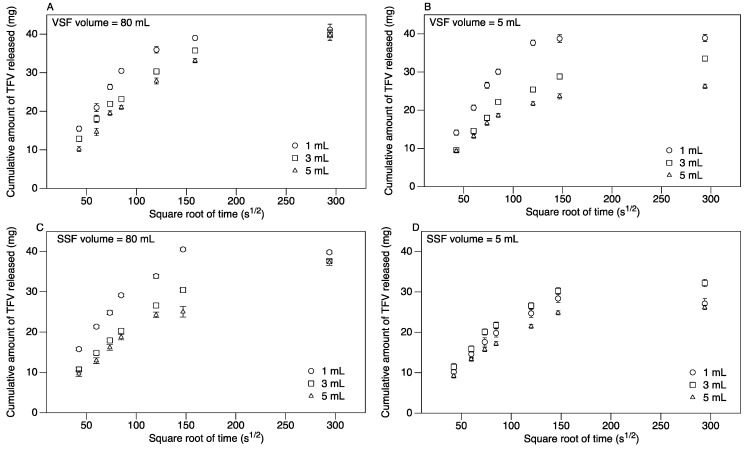
The effect of size on the diffusion of TFV from spherical carrageenan suppositories in: (**A**) 80 mL of vaginal simulant fluid (VSF); (**B**) 5 mL of VSF; (**C**) 80 mL of semen simulant fluid (SSF); and (**D**) 5 mL of SSF. The release of TFV by diffusion from suppositories 1, 3 and 5 mL loaded with 40 mg TFV each was studied for 24 h. Plotted are the mean and standard error (*n* = 7) of the cumulative amount of TFV released.

For diffusion into 80 mL VSF, 50%–75% of the encapsulated TFV was released within the first 2 h (depending on suppository volume), and over 80% was released within 6 h (Figure 3A). All of the TFV within the ovule was released within 24 h, irrespective of size (Figure 3A). For diffusion into 5 mL VSF, 45%–75% of the encapsulated TFV was released within the first 2 h (depending on suppository volume), as shown in Figure 3B. Over 95% of TFV in 1 mL ovules was released within the first 24 h (Figure 3B). At 24 h, 80% and 65% of the encapsulated TFV was released from the 3 and 5 mL spheres, respectively. The diffusion of TFV from the 3 and 5 mL sizes plateaued at 24 h, with minimal changes in TFV diffusion over the next 24 h (data not shown). However, replacing the VSF in the tubes with fresh VSF at 24 h did stimulate the release of additional TFV, indicating insufficient sink conditions (data not shown), suggesting that replenishment of vaginal fluid physiologically would enable complete release.

For diffusion into 80 mL SSF, 45%–70% of the encapsulated TFV was released within the first 2 h (depending on suppository volume), and 60%–80% was released within 6 h from 3 to 5 mL-sized suppositories. All of the TFV is released from 1 mL-sized suppositories in 6 h (Figure 3C). For 3 and 5 mL-sized suppositories, over 95% of the TFV within the ovules was released within 24 h (Figure 3C). For diffusion into 5 mL SSF, 40%–50% of the encapsulated TFV was released within the first 2 h (depending on the suppository volume), as shown in Figure 3D. At 24 h, 70%–80% of the encapsulated TFV was released from the spheres (depending on suppository volume). Similar to the VSF studies above, the TFV release reached a plateau at 24 h, and replenishing with fresh SSF stimulated further release of TFV.

The initial TFV release profile in VSF and SSF (0–2 h) showed a linear relationship between the cumulative release of TFV and the square root of time. The slope of the linear portion of the line (0–2 h) was used to calculate the rate of TFV diffusion (Table 1). For release into VSF and SSF, there was a significant difference in the rate of diffusion between the three sizes in the first 2 h for both the 80 and 5 mL diffusion curves (Table 1). For release into 80 mL of dissolution medium, there were significant differences between the rates of diffusion in VSF *versus* SSF for 3 and 5 mL, whereas in 5 mL of dissolution medium, the differences in the rate of diffusion in VSF *versus* SSF were not significant, except for 1 mL ovules (Table 1). In VSF, the release in a 5 mL volume is significantly lower than 80 mL for 3 and 5 mL suppositories. In SSF, the release rate in 5 mL volume is significantly lower than 80 mL for the 1 mL suppository, and with the 3 mL suppository, the rate is slightly higher for a 5 mL SSF volume.

**Table 1:** Size influences the rate of TFV diffusion from semisoft carrageenan suppositories. Calculated *in vitro* release rates for TFV into VSF and SSF from the three different sizes of ovules.

Suppository Size	LSM release rate in mg/(s^½^)			
	80 mL VSF	5 mL VSF	80 mL SSF	5 mL SSF
1 mL	0.345 ^a,A,1^ ± 0.002	0.356 ^x,A,6^ ± 0.005	0.341 ^d,F,1^ ± 0.002	0.238 ^g,G,7^ ± 0.003
3 mL	0.282 ^b,B,2^ ± 0.002	0.257 ^y,C,8^ ± 0.005	0.241 ^e,H,3^ ± 0.002	0.256 ^h,I,8^ ± 0.003
5 mL	0.251 ^c,D,4^ ± 0.002	0.217 ^z,E,9^ ±0.005	0.219 ^f,J,5^ ± 0.002	0.210 ^k,J,9^ ± 0.003

Lower case letters denote statistically significant differences in the diffusion rates across the three suppositories sizes for the same VSF/SSF volume. Upper case letters denote statistically significant differences in the diffusion rates between the two volumes of dissolution medium (VSF/SSF) for the same suppository size. Numbers denote statistically significant differences in the diffusion rates between the VSF and SSF for the same volume and same suppository size.

## 4. Discussion

Microbicides are a promising means of HIV prevention, especially for women, who carry more than 50% of the AIDS burden in some parts of the world [1]. Several different forms of microbicides are currently being researched and in clinical trials, but no commercial microbicide products have been introduced to date; and there are shortcomings with existing prototypes (e.g., leakage with liquid gels [10]). Hence, there is continuing need to explore and develop new microbicide prototypes preclinically.

We have developed semisoft vaginal suppositories from carrageenan and conducted various studies to determine the physical properties of the suppositories that drive user acceptability [13,28,29]. To further their development as functional microbicides, drug release studies in water, VSF and SSF were conducted using suppositories loaded with the antiretroviral drug tenofovir (TFV). Due to the charged nature of TFV and carrageenan, it is critical to ensure that TFV is successfully released into the medium and not bound to the matrix. To better mimic the vaginal environment, release studies were also conducted in 5 mL of VSF to closely represent the actual volume of vaginal fluids. The dissolution studies in water were conducted to characterize drug release in a neutral medium and to quantify the loading efficiency of the ovules. Additionally, this product is designed for use prior to and during coitus; hence, it needs to effectively release TFV, even in the presence of semen. During diffusion studies with a large volume of VSF and SSF (80 mL), there is a difference in the initial rate of diffusion based on the size of the suppositories; however, almost all of the encapsulated TFV diffuses out of the suppositories in 24 h. For dissolution in 80 mL of medium, the higher rates of diffusion in VSF as compared to SSF can be explained by the greater solubility of tenofovir (pK_a_ 3.8 (acidic) and 6.7 (basic)) in a medium of pH 4–6.5 (data not shown). The rate of drug diffusion from a delivery matrix partly depends on the concentration of drug in the saturated layer around the matrix, which depends on the solubility of the drug [38]; hence, tenofovir diffuses faster out of the carrageenan matrix into VSF as compared to SSF. The release rates in 5 mL of VSF are lower than in 80 mL, due to the saturation of the VSF medium surrounding the suppositories. For diffusion into 5 mL of VSF and SSF, there is a significant difference in the initial rate (0–2 h) of diffusion from the three sizes, as well as the total amount of TFV released into the medium at 24 h without fluid replacement. Notably, the initial diffusion rates in 5 mL of VSF and SSF are not significantly different (except for the 1 mL size). This ensures that the diffusion of the drug continues at the same rate during coitus and in the presence of semen; thus providing uninterrupted protection to women. In the case of 1 mL sized ovules, the diffusion rate in SSF is slower than in VSF; however, the TFV continually diffuses out of the ovules, offering protection.

Although the favored shape from consumer tests was a long oval, we used spherical suppositories for the drug release studies described here to better model release. In VSF and SSF, the tenofovir is released from the suppositories by diffusion, and the release rate depends on the ratio of the surface area to the volume of the shape (Equation (1)). The volume for both of the shapes is the same, and the surface area is slightly higher for a long oval (10.5 cm^2^), as compared to a sphere (9.08 cm^2^). Hence, the release rate for a long oval can be estimated to be slightly higher compared to a sphere.

We have conducted focus groups previously [13] to determine favorable physical attributes, as well as use parameters (such as the duration and frequency of use) that drive women’s willingness to try this product. Some women indicated that they would prefer a fast acting product that could be inserted prior to coitus and be effective for a couple of hours, whereas some women preferred a coitally-dissociated product. These preferences are greatly influenced by the women’s age, relationship status and frequency of sex. Hence, the current drug dissolution studies help characterize release, as well as study parameters that affect diffusion rates, which can be modulated to design a favorable product.

Our experimental setup has the limitation of inadequate sink conditions, resulting from the saturation of TFV in the medium surrounding the spheres. Replenishing the tube with fresh VSF/SSF stimulates the diffusion of additional TFV. The average amount of vaginal fluid present in the vagina at any given time is 0.5–1 mL [39], and this volume significantly increases during ovulation and sexual stimulation ([40,41] as cited by [36]); however we used 5 mL for the vaginal simulation study to maintain at least a 1:1 ratio of dissolution medium volume to suppository volume. The tenofovir drug must diffuse out of the suppository into the vaginal lumen, where it is absorbed into the vaginal tissue and, eventually, into the systemic circulation. Thus, this limitation can be overcome with experimental conditions that help maintain sink conditions by avoiding TFV saturation to better mimic the vaginal environment. The vaginal discharge in women is composed of several components, such as vulvar secretions, cervical mucus and endometrial fluid, and its amount varies based on several factors, such as menstrual cycle, hormone levels and sexual arousal [42]. Due to physiologic processes, there is regular flushing of the fluids in the vagina, which may help maintain a concentration gradient to allow TFV diffusion into the vaginal lumen. The other consideration is that TFV prophylaxis works by diffusing into the vaginal walls, which may help maintain a concentration gradient across the suppository, vaginal lumen and vaginal tissue [43]. During coitus, there is the addition of the ejaculate volume. To make results obtained in VSF and SSF comparable, we chose a constant 5 mL volume.

The carrageenan suppositories were originally conceptualized to break down in the VSF and be eliminated with vaginal mucous secretions. The appearance of the carrageenan matrix closely mimics vaginal mucous secretions, which would facilitate covert use. However, the current generation of prototypes does not break down in VSF or SSF. While this may be acceptable in some circumstances, for example, where covert use is not required, efforts to formulate suppositories that will erode are underway.

## 5. Conclusions

We have successfully demonstrated that at least 40% of the TFV diffuses out of the suppositories within the first two hours, irrespective of size and volume in both vaginal and semen simulant fluid in this proof of concept study, suggesting that further development of the carrageenan-based semisoft suppository prototypes is merited.
